# The Longitudinal Communication Curriculum at Leipzig University, Medical Faculty – implementation and first experiences

**DOI:** 10.3205/zma001454

**Published:** 2021-03-15

**Authors:** Anja Zimmermann, Christoph Baerwald, Michael Fuchs, Christian Girbardt, Heide Götze, Gunther Hempel, Kai von Klitzing, Daisy Rotzoll

**Affiliations:** 1 University of Leipzig, Medical Faculty, LernKlinik Leipzig, Skills and Simulation Centre, Leipzig, Germany; 2 University of Leipzig, Medical Faculty, Centre for Didactics in Medicine, Leipzig, Germany; 3 University of Leipzig Medical Centre, Medical Department III – Endocrinology, Nephrology, Rheumatology, Leipzig, Germany; 4 University of Leipzig Medical Centre, Department of Otorhinolaryngology, Head and Neck Surgery – Division of Phoniatrics and Audiology, Leipzig, Germany; 5 University of Leipzig Medical Centre, Department of Ophthalmology, Leipzig, Germany; 6 University of Leipzig, Medical Faculty, Division of Medical Psychology and Medical Sociology, Leipzig, Germany; 7 University of Leipzig Medical Centre, Department of Anaesthesiology and Intensive Care, Leipzig, Germany; 8 University of Leipzig Medical Centre, Department of Paediatric Psychiatry, Psychotherapy and Psychosomatics, Leipzig, Germany

**Keywords:** communication skills, curriculum development, simulated patient

## Abstract

**Purpose: **Communication skills are an essential instrument for building a sustainable patient-doctor-relationship for future doctors. They are learnable and teachable. The learning should be facilitated with the help of a longitudinal curriculum, which is planned at Leipzig University.

**Project: **At the Medical Faculty of Leipzig University, the Longitudinal Communication Curriculum is established since 2016/17. Up to now, the curriculum consists of four parts in which students repeatedly practise their communication skills in curricular and extracurricular courses. Several formats help to teach an integrated learning of communication and physical examination skills. Assessment of communication skills is also performed. Curricular implementation is accompanied by concomitant evaluation.

**Results: **Three parts of the curriculum already have taken place. Students report an increase in communication skills. Students rate the units as instructive and helpful. The assessment of communication skills occurs in two clinical practical examinations (OSCEs). Together with summative assessment a formative feedback was implemented. Students judge this practice as highly positive.

**Discussion: **The curriculum is part of undergraduate medical education in Leipzig. It would be beneficial to add another simulated patient encounter, as well as interprofessional units. Student questionnaires will be evaluated and results will help to develop the curriculum.

**Conclusion: **Consolidation of the curriculum accompanied by evaluation and adaption of content can help to assure the quality of the curriculum. Additional professions and study units shall be integrated in the Longitudinal Communication Curriculum in the future.

## Zusammenfassung

**Zielsetzung:** Kommunikative Kompetenzen gelten für angehende Ärztinnen und Ärzte als wichtiges Instrument zum Aufbau einer tragfähigen Arzt-Patient-Beziehung und sind lehr- und lernbar. Die Vermittlung sollte möglichst über longitudinale Curricula erfolgen. Dies wird an der Universität Leipzig angestrebt.

**Projektbeschreibung: **An der Medizinischen Fakultät der Universität Leipzig wird seit 2016/17 ein longitudinales Curriculum für kommunikative Kompetenzen implementiert. Das Curriculum ist aktuell in vier Abschnitte geteilt, in denen Studierenden in curricularen und extracurricularen Veranstaltungen unterschiedliche Gesprächsführungskompetenzen wiederholt vermittelt werden. Dies erfolgt über verschiedene Lehrformate, wobei besonderer Wert darauf gelegt wird, dass eine integrierte Vermittlung von kommunikativen und klinischen Fertigkeiten erfolgt. Auch die Prüfung verschiedener kommunikativer Kompetenzen soll implementiert werden. Die Implementierung des Curriculums wird von einer Evaluation begleitet.

**Ergebnisse: **Drei Abschnitte des Curriculums wurden bereits implementiert. Studierende berichten einen Zuwachs an Gesprächsführungskompetenzen und erleben die einzelnen Elemente des Curriculums als lehr- und hilfreich. Kommunikative Kompetenzen werden in zwei klinisch-

praktischen Prüfungen erhoben. Dabei konnte neben der summativen auch eine formative Prüfung via Feedback eingeführt werden. Dies wird durch die Studierenden positiv bewertet.

**Diskussion: **Das Curriculum ist inzwischen Bestandteil des Leipziger Humanmedizinstudiums. Eine Ergänzung der bisher bestehenden Einheiten insbesondere um ein weiteres Gespräch mit Simulationspersonen sowie interprofessionelle Einheiten erscheint sinnvoll. Die Befragung der Studierenden soll ausgewertet und die Ergebnisse in die Entwicklung des Curriculums einbezogen werden.

**Schlussfolgerung: **Eine Verstetigung des Curriculums einhergehend mit einer Evaluation und Anpassung der Inhalte kann zur Qualitätssicherung beitragen. Weitere Fächer und Studienabschnitte sollen zukünftig Bestandteil des longitudinalen Kommunikationscurriculums werden.

## 1. Introduction

Communication skills are essential for building a sustainable patient-doctor-relationship. These skills are teachable and learnable [1], [2], [3]. Feedback is an important tool for continuous development of individual skills [4], [5]. Furthermore, professional feedback to examinees can support the development of clinical competence in assessment situations [6].

Working with simulated patients (SPs) makes it possible to practice physical examination techniques and patient contacts repeatedly. Such contacts can easily be scheduled and the use of SPs in assessment situations is well established [7], [8].

Teaching and assessing communication and social skills with the introduction of longitudinal curricula is widely promoted nationally and internationally [2], [3], [9], [http://www.nklm.de]. Aims of such curricula are competence orientation, focus on patient needs, interprofessional education and curricular alignment of learning objectives in medical education [10].The CanMEDS framework defining medical competencies is a well-established basis for the development of a longitudinal communication curriculum [11].

In 2015, 10 out of 39 German medical faculties had a longitudinal communication curriculum implemented in their medical curriculum [12]. Most skills in communication are taught and assessed during the first three undergraduate years of medical education. Various methods such as role play, small group work and SP encounters are used [13], [11], [14].

Since 2016, a structured longitudinal curriculum in communication is being integrated into the existing medical curriculum at the Medical Faculty in Leipzig, combining theoretical basics in communication with clinical training. To support this implementation, a SP programme was initiated to support educational strategies in all clinical disciplines involved.

## 2. Project

The longitudinal communication curriculum was developed by a project group of preclinical (medical psychology and medical sociology) and clinical colleagues (ophthalmology, internal medicine, anaesthesiology, otolaryngology and child psychiatry), as well as medical students. The director of the Department of Paediatric Psychiatry, Psychotherapy and Psychosomatics at University of Leipzig Medical Centre and the medical director of the Skills and Simulation Centre LernKlinik Leipzig lead the group.

The curriculum starts in the 3^rd^/4^th^ semesters and continues throughout the entire undergraduate medical education.

Communication skills are taught repeatedly. During the different parts of the Longitudinal Communication Curriculum various learning objectives are in focus. The roles Communicator and Team Manager of CanMEDs [9] serve as starting point.

For implementation, learning objectives out of the existing curriculum of these courses were chosen: topics that require clinical practical skills, topics that include or have clinical practice planned and topics that are interdisciplinary.

**Figure 1:**
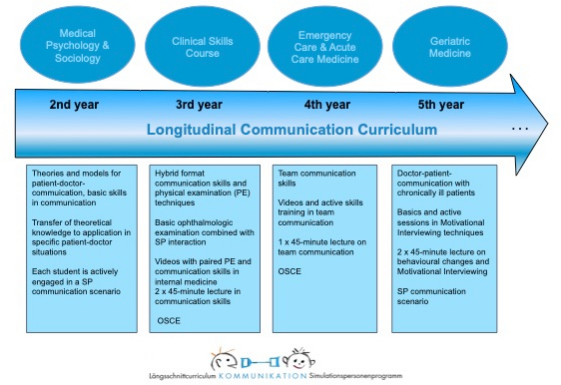
Longitudinal Communication Curriculum at Leipzig University, Medical Faculty. Abbreviations: OSCE (objective structured clinical examination), SP (simulated patient)

The aim is an integration of the existing curriculum into the reformed course of studies with a longitudinal, interdisciplinary teaching of communication and social skills (for the curriculum see figure 1 (Fig. 1)).

For fostering curricular implementation a centrally coordinated SP programme is established.

The implementation of the Longitudinal Communication Curriculum and the SP programme is funded by the Saxon State Ministry for Science, Culture and Tourism (SMWK) since winter semester 2016/17. The Longitudinal Communication Curriculum as well as the SP programme are meanwhile part of the curriculum in Leipzig. It is described in the following.

### 2.1. Part I: 2nd year of medical studies

The Longitudinal Communication Curriculum starts in the second academic year within the communication course of the division of medical psychology and medical sociology, which is based on the COMSKIL model (COMSKIL, [15]). Basic communication content is taught in the course. In addition to imparting communication content over two semesters, the students practice building up a patient-doctor relationship in SP encounters. Groups of 10 students each experience 11 different SP encounters. Courses are moderated by faculty members of the Division of Medical Psychology and Medical Sociology as well as by peer-student tutors from the LernKlinik Leipzig who are didactically and professionally trained.

**Figure 2:**
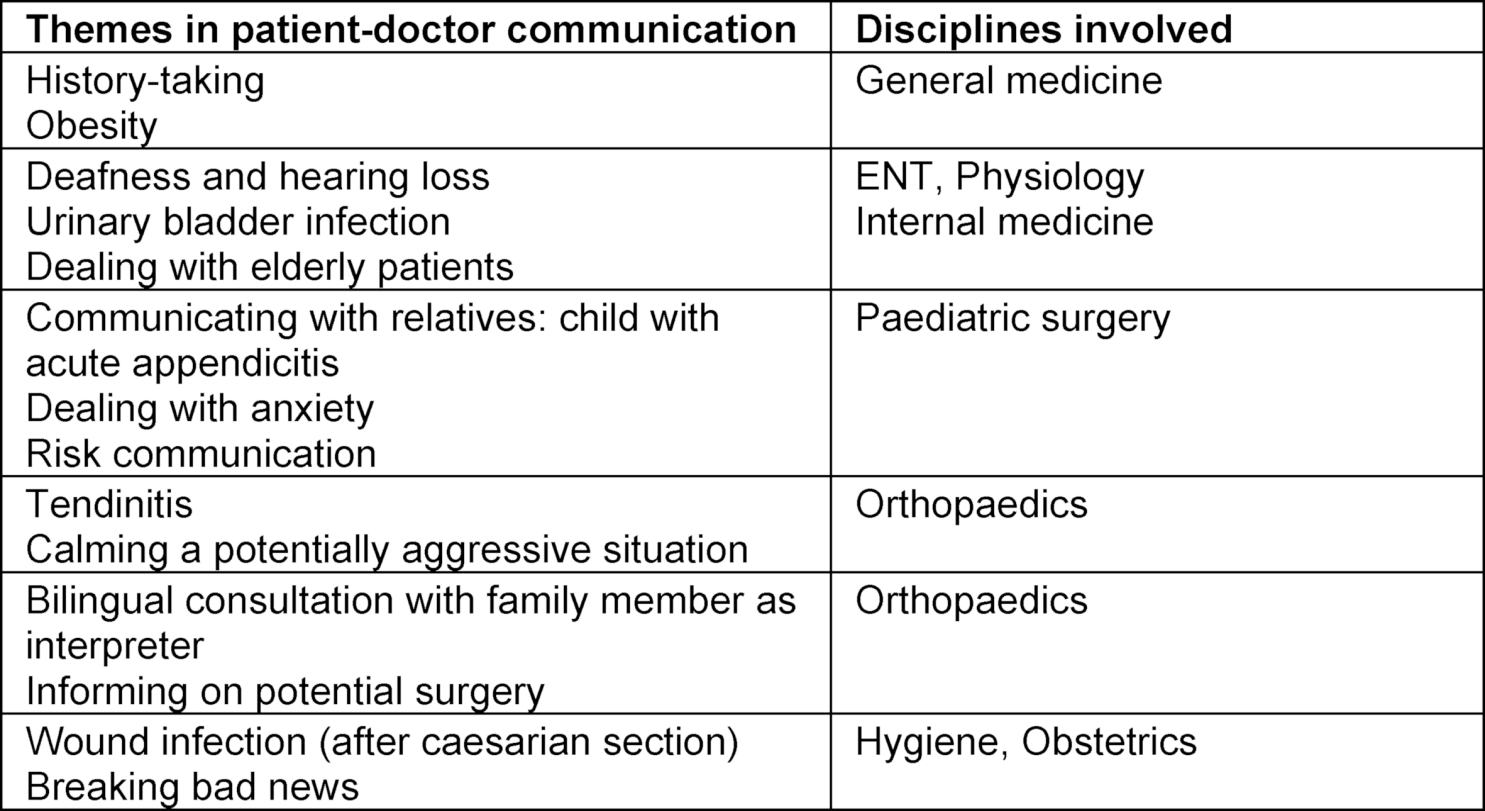
Linking preclinical to clinical years – SP communication scenarios as part of the Longitudinal Communication Curriculum for 2^nd^ year medical students

One communication unit including preparation and feedback lasts 50 minutes. The topics are thematically connected to clinical scenarios (see figure 2 (Fig. 2)). These examples are edited in a way students themselves can prepare for the course. The topics furthermore are part of the communication course itself. Basic knowledge is imparted to students prior to the SP encounters in order to be able to deal with different situations. Every student is involved in a SP encounter and receives feedback from this SP, the participating peers and the moderating faculty or peer-student tutors. During the other SP encounters the students have the possibility of participating while observing their peers. At the end, they give a structured feedback to their peers.

#### 2.2. Part II: 3rd year of medical studies

The so-called Clinical Skills Course takes place in the 5^th^ semester where students learn physical examination techniques. Additionally, basic skills in communication are repeated and complemented. This takes place in two lectures focusing on patient-doctor communication in internal medicine. Accompanying these two lectures students watch videos on clinical skills and medical communication followed by a worksheet preparation. The videos are available online. Students have to deal with a defined task. They have to recognize “mistakes” both in medical communication and in clinical skills. For problem-solving and exam preparation, positive video examples are available after task completion.

In the Clinical Skills Course for ophthalmology, students can participate in SP encounters. The previously learned basic ophthalmologic examination techniques have to be applied, while dealing with communication goals in a difficult communication situation (patient with suspicion of multiple sclerosis). Students receive or give structured feedback on the dimensions of medical communication (Berlin global rating, see 2.6) and clinical skills. A communication unit lasts 30 minutes, including preparation, SP encounter and feedback. 

The Clinical Skills Course is assessed by an objective structured clinical examination (OSCE). Hereby students are assessed not only in clinical skills but also in communication skills at one out of five stations since 2018.

For OSCE preparation, extracurricular courses are available at the LernKlinik Leipzig. Students can prepare for examination in clinical and communication skills in a 120-minute course involving SPs. Every participating student leads a conversation including a physical examination of the SP. Concomitantly a structured feedback is offered. These courses are held by peer-student tutors of the LernKlinik Leipzig.

#### 2.3. Part III: 4th year of medical studies

Lacking communication skills, rather than medical skills, can lead to false decisions especially in critical situations [16]. This should be taken into account and trained throughout the medical curriculum, particularly in cooperation with interprofessional teams. In Leipzig, the theoretical background for team communication and teamwork is based on the framework of crisis resource management (CRM, [16]). Within the curriculum, students in the 7th semester, experience and learn team communication during the problem-based learning (PBL) course on emergency care and acute care medicine.

A 45-minute lecture introduces the students to the basics of CRM. They can then apply this theoretical foundation they have learned in practice during the emergency room (ER) management.

Students prepare for this session by watching a video (negative example) and answering questions. The task is to recognize basics of CRM and transfer them to practice. During training, video debriefing is followed by a positive-example demonstration performed by medical faculty and students in their final year of medical school during their hospital clerkship. Finally, the students themselves perform an active role in a predefined trauma room scenario. Learning objectives comprise knowledge about different roles in the ER and importance of teamwork and safe communication. Each role-play ends with a short debriefing. The complete unit lasts 90 minutes.

The entire PBL session lasts four weeks and is assessed via OSCE. Since 2019, team communication skills are assessed in addition to emergency skills.

Students are offered extracurricular courses for OSCE preparation. These are offered by peer-student tutors of the LernKlinik Leipzig. Two such courses are hybrid courses focusing on team communication and emergency skills. These courses last 60 minutes each.

#### 2.4. Part IV: 5th year of medical studies

So far, the final unit of the curriculum planned will be integrated into the PBL course on medicine of the elderly in the 10^th^ semester, prior to the final year in medicine comprising the hospital clerkship. The aim is to primarily introduce the topic of behavioral change with techniques of Motivational Interviewing (MI) [17]. For this purpose, a 45-minute lecture is planned, starting in the summer term of 2020. Students can work on a PBL case dealing with behavioural change. SP encounters involving MI are to take place in a pilot phase. In these the learned knowledge can be put into practice.

#### 2.5. SP programme

Since 2017, a SP programme is established at the Medical Faculty of Leipzig University. So far, it consists of 38 trained participants. SPs are recruited continuously and prepared for SP roles and giving a structured feedback after a patient-doctor-interview. For this purpose, role scripts are provided for the SPs. They are prepared by the SP programme coordinator in close agreement with the individual medical disciplines. Furthermore, there are trainings in role-play and feedback for SPs. Each role is trained repetitively before the respective assignment (usually once per semester), with focus on the presentation of specific symptoms and medical history. Situations that may take place in the SP-medical student encounters are trained with the help of peer-student tutors. For feedback preparation, SPs receive a theoretical introduction and the opportunity to exchange their experiences. After the encounter rehearsal, SP feedback is performed, followed by a wrap-up by all participants of the training rehearsal.

For OSCE assessment, SPs are trained as standardized patients. The OSCE is completed by a structured, formalized SP feedback, followed by a feedback of the faculty assessor.

#### 2.6 Aims: the Berlin global rating scale and feedback

##### 2.6.1. The Berlin global rating scale as framework of the longitudinal communication curriculum

The Berlin global rating scale (BGR) is used as framework for evaluating patient-doctor encounters [18], [19]. This scale was selected due to its simple structure and easy applicability.

Students are introduced to the BGR during COMSKIL courses in their second year of medical education. They get to know the BGR as a global instrument for evaluating patient-doctor encounters by focusing on four dimensions: empathy, structural, verbal and nonverbal expression. In the Longitudinal Communication Curriculum, the BGR is used for self- and peer-assessment. Accordingly, the scale is also used as the basis for communication skills assessment in the OSCE. Through continuous use, students have the opportunity to appreciate this scale as a global instrument for evaluation and improvement of their communication skills. 

##### 2.6.2. Feedback as recurrent element throughout the curriculum

Feedback is used for giving students a structured and immediate response to their ongoing learning [4], [5], [6], [20]

After every SP encounter, feedback on communication skills is given. Furthermore, students themselves practise giving feedback. Every SP encounter is followed by a SP feedback, a peer feedback and a feedback from faculty. 

During the OSCE, feedback is also provided. In both OSCEs, after the 5^th^ and 7^th^ semester respectively, students receive feedback from their examiner after each station within the OSCE. At stations with SP involvement, students also receive a formalized and structured SP feedback. Students receive feedback on (team) communication as well as clinical skills. Examiners are trained to give structured feedback. This feedback gives the students the opportunity to learn which specific clinical or communication skills they need to work on.

**Figure 3:**
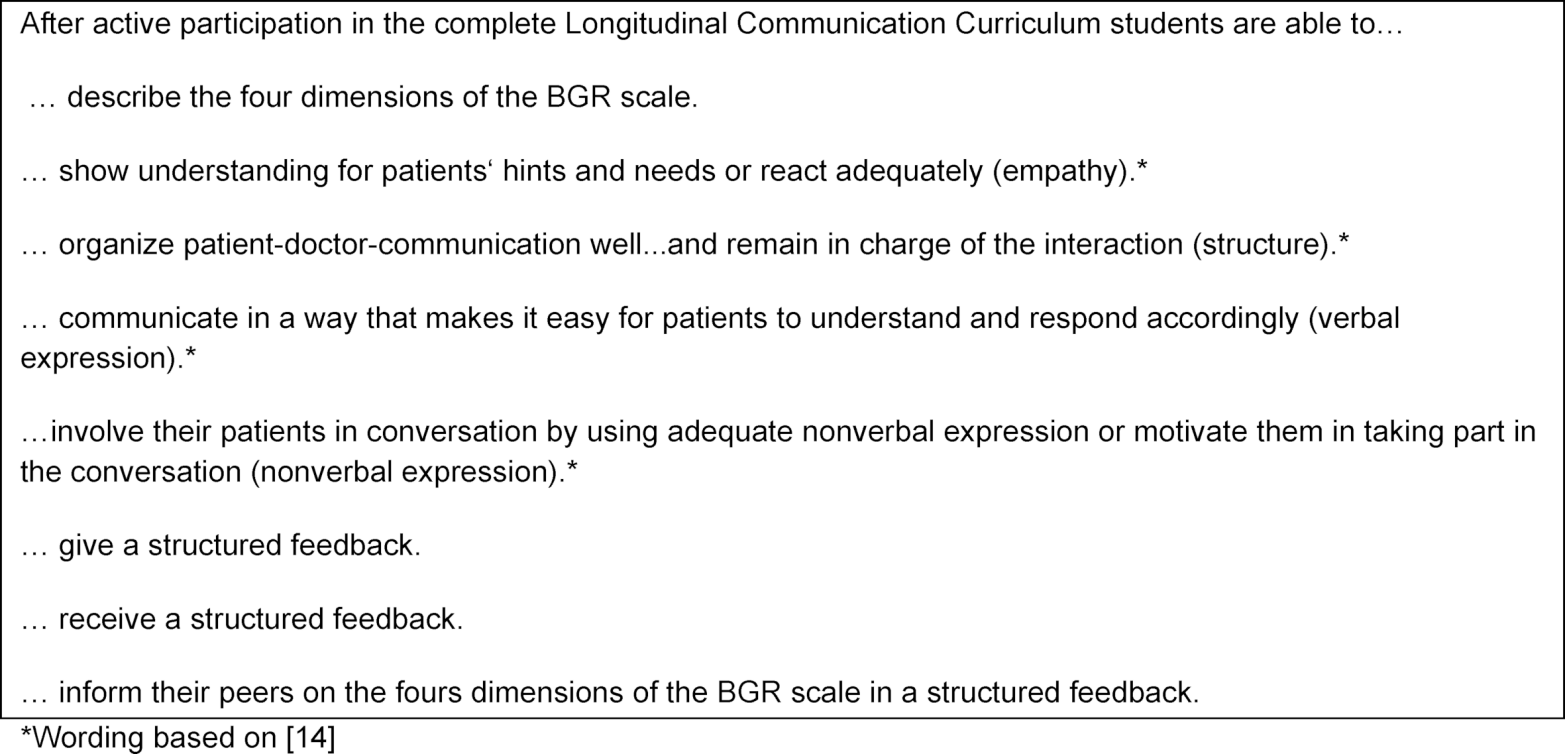
Longitudinal Communication Curriculum: overall learning objectives. Abbreviation: BGR (Berlin Global Rating)

BGR and feedback are recurrent themes throughout the entire curriculum. In figure 3 (Fig. 3), the learning objectives of the complete Longitudinal Communication Curriculum are summarized.

## 3. Results

**Figure 4:**
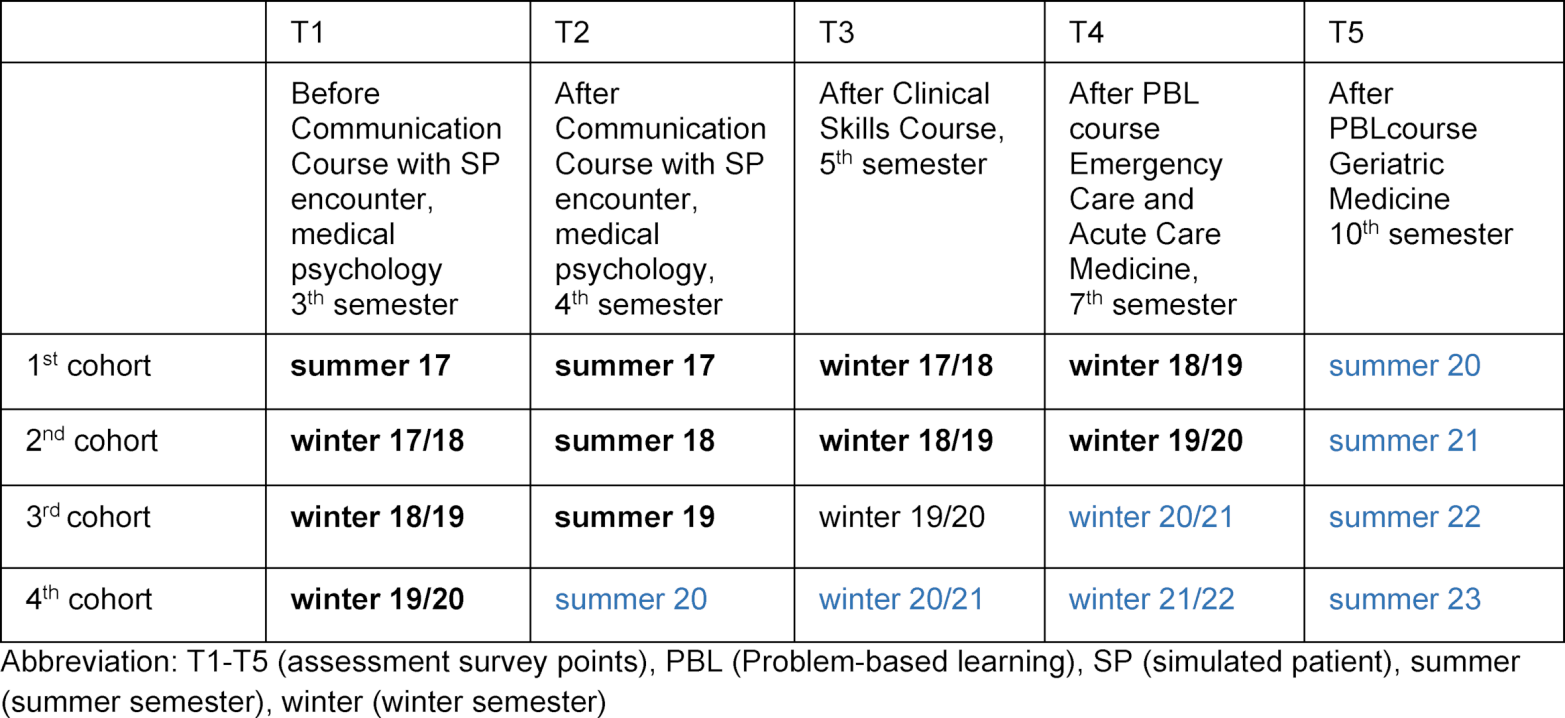
Assessment survey points (T1 – T5) with already obtained data (bold marking), future assessment points for cohorts (in blue).

The implementation of the curriculum is continuously evaluated. Students answer mainly paper-based questions (to increase the return rate). Questionnaires are distributed via EvaSys^®^ (Evasys GmbH, 2017-2019, Lüneburg). The first cohort will have completed the curriculum by the end of summer term 2020. Students are all asked to take part in five questionnaire-based assessments over time (T1-T5). Data have been collected up to the 4^th^ cohort (see figure 4 (Fig. 4)).

Data on attitude towards communication are collected via the Communication Skills Attitude Scale [21], the relevance of empathy in the patient-doctor-relationship via the Jefferson Scale of Physician Empathy, student version [22], and data on personal communication skills are recorded via an adaptation of the BGR [18], [19].

Furthermore, students rate which changes in communication skills they observe on themselves. The learning situations of the Longitudinal Communication Curriculum are evaluated by the students as well (Likert scales).

We report the results of students’ evaluation on the first three assessment points of the curriculum for cohorts 1 and 2 as well as the 4^th^ assessment point for cohort 1.

Data were analyzed using IBM^®^ SPSS^®^ Statistics Version 24.

### 3.1. Ethics approval and participation consent

Students were informed about the study. Written consent was obtained at the 1^st^ assessment point (T1). At every further assessment point students were informed that their data are part of a study. Data were collected anonymously. This study received an approval of the ethics committee of the Medical Faculty, University of Leipzig (149/17 – ek).

#### 3.2. Curriculum evaluation by cohorts one and two

After each course unit, students evaluated the respective course and the overall Longitudinal Communication Curriculum.

At the 2^nd^ assessment point (T2) we obtained data from n=445 students (mean=22 years, 67% women, 33% men). At the 3^rd^ assessment point (T3), data of n=385-548 students are available (several questions were only partly answered, mean age=23 years, 66% women, 34% men). From the 4^th^ assessment point (T4), data of n=72 students of the first cohort are available (mean age=24 years, 65% women, 35% men).

When comparing data from cohort 1 and 2, no differences can be seen concerning self-assessed communication skills, age or gender. Students of both cohorts had a SP encounter between T1 an T2. The Clinical Skills Course, SP encounter in ophthalmology and video analyses in internal medicine took place between T2 and T3.

**Figure 5:**
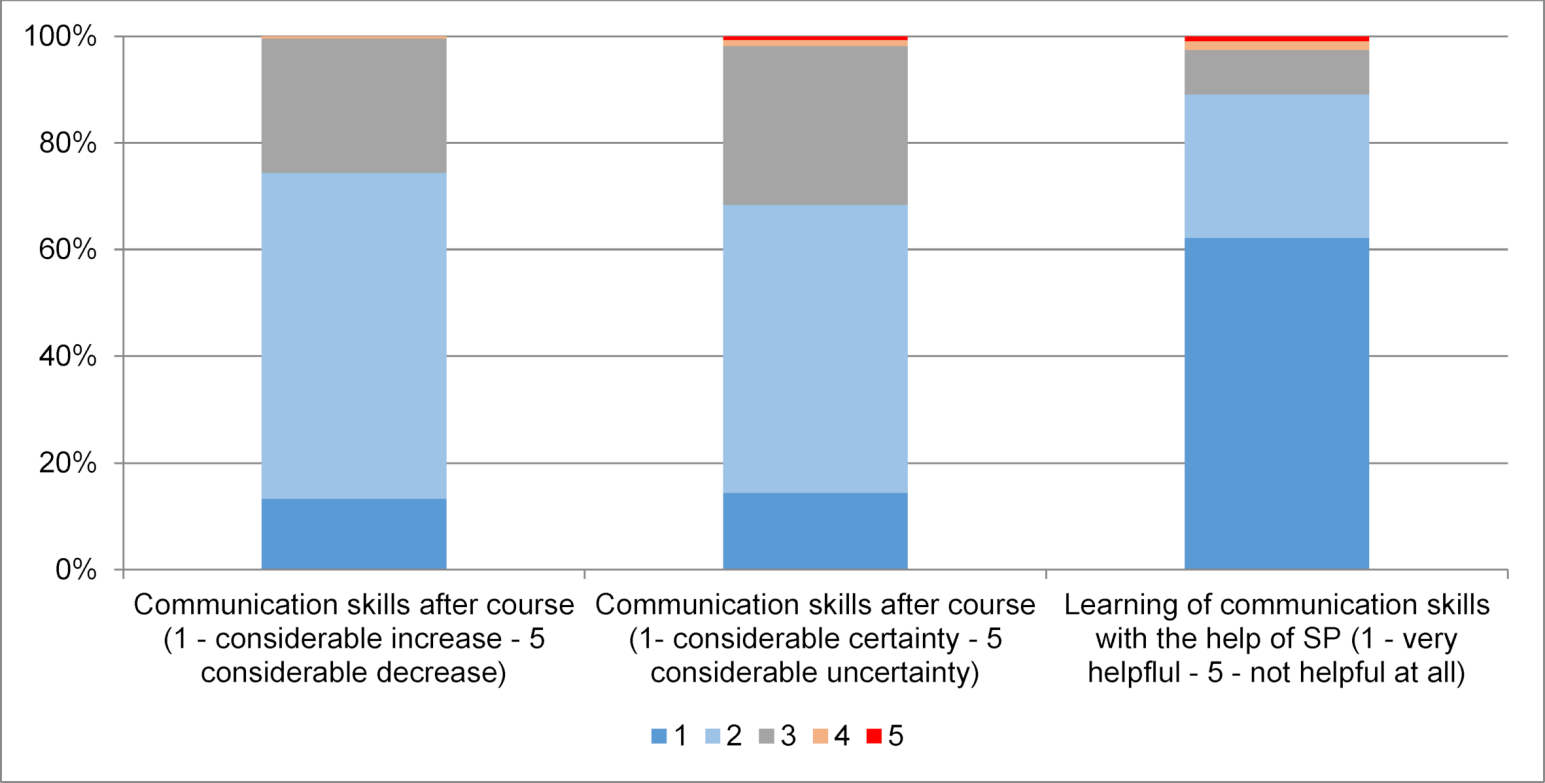
Judging of communication skills after communication course 4^th^ semester with simulated patients (SP), 2^nd^ assessment point, cohorts 1 and 2, n=445, mean age=22 years, 67% women, 33% men.

In T2, students report an improvement in communication skills and experience SPs as helpful for development of communication skills (see figure 5 (Fig. 5)).

**Figure 6:**
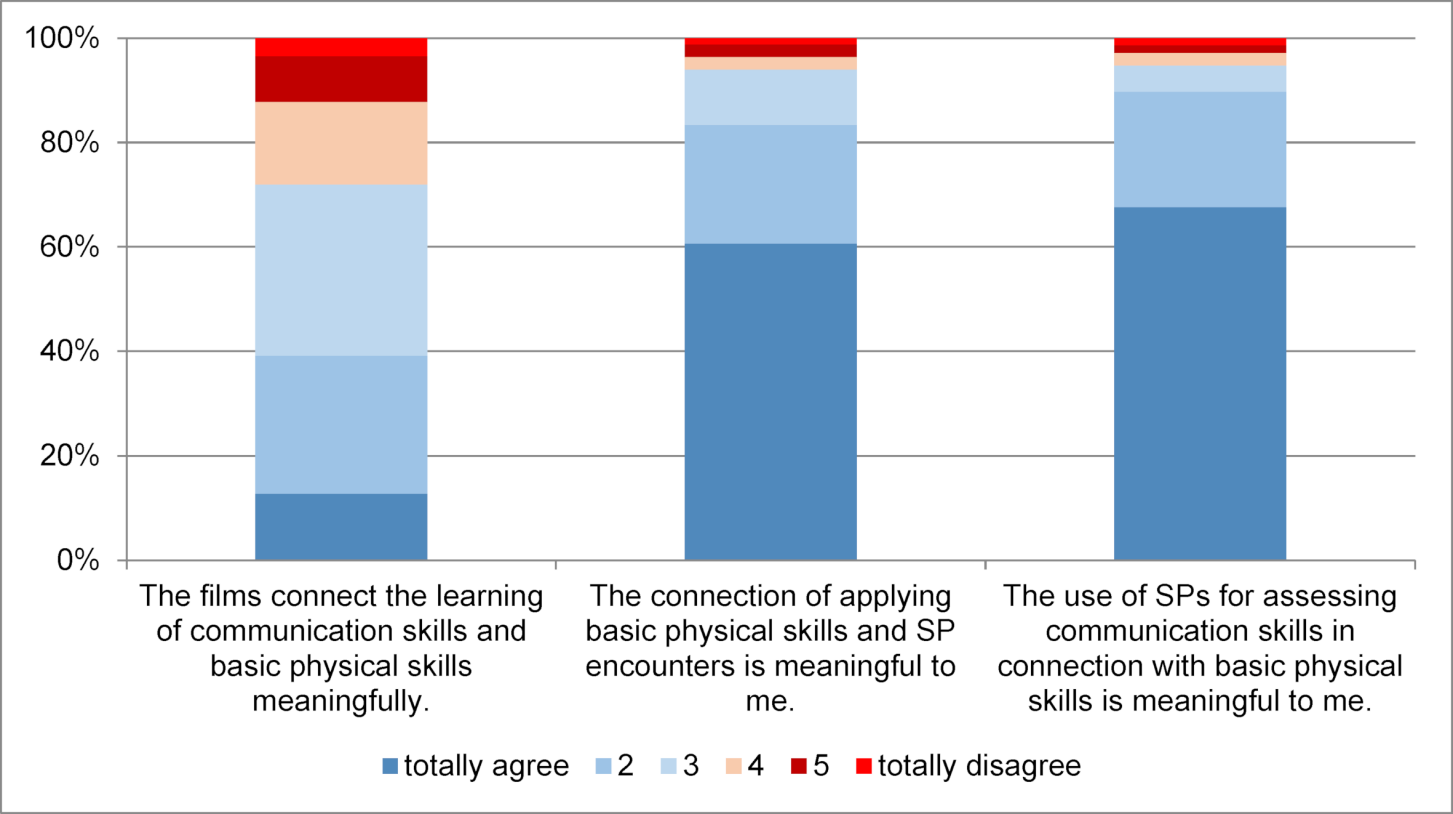
Judging of films and simulated patient (SP) encounters in the Clinical Skills Course, 3^rd^ assessment point, cohorts 1 and 2, n = 385/422/548, mean age=23 years, 66% women, 34% men.

**Figure 7:**
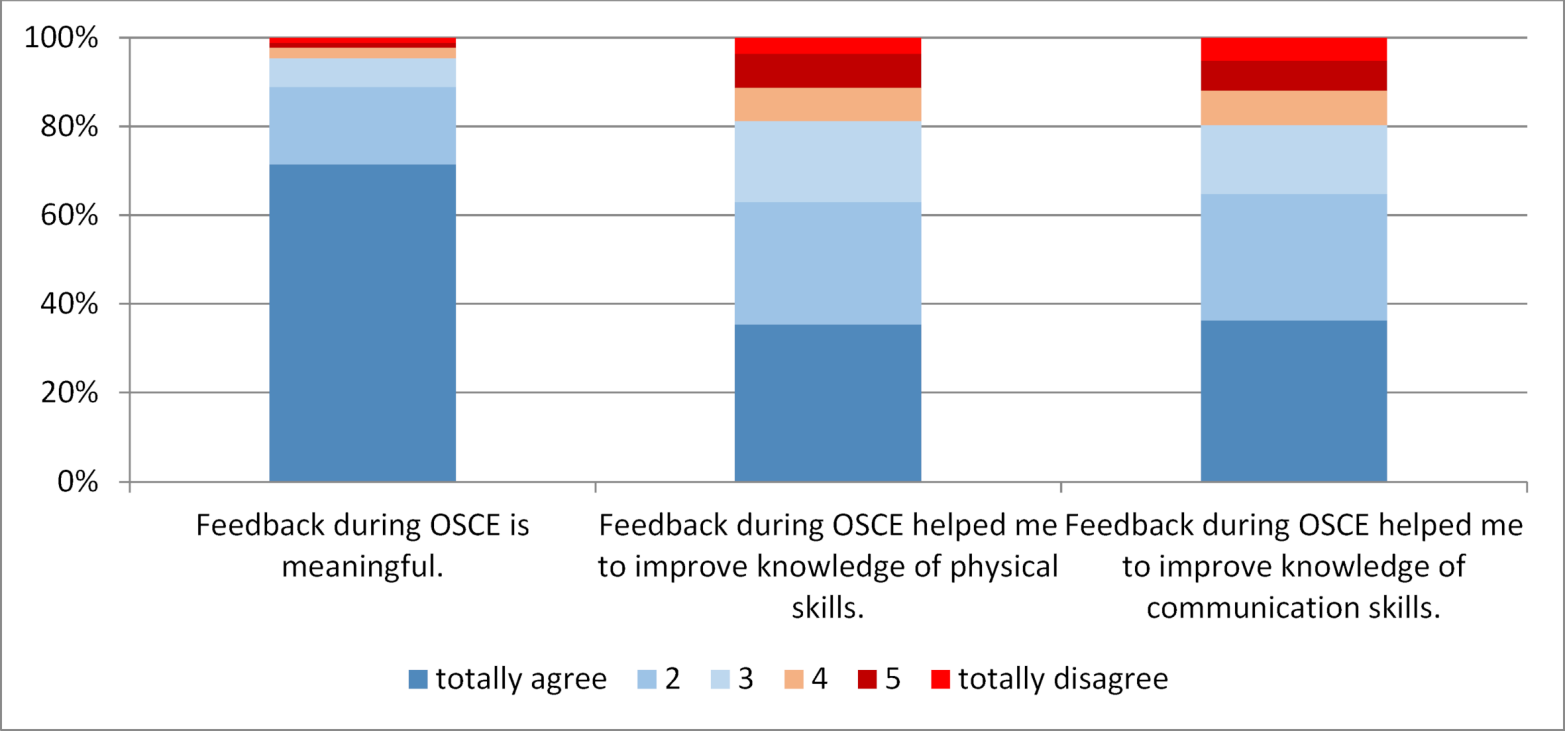
Judging of feedback during OSCE, 3^rd^ assessment point, cohorts 1 and 2, n=548, mean age=23 years, 66% women, 34% men.

After the Clinical Skills Course (T3) in the 5^th^ semester, both video analysis and SP encounter were regarded as a valuable combination of learning the use of communication skills and clinical examination skills (see figure 6 (Fig. 6)). Receiving feedback from SPs during OSCE assessments is also considered as useful (see figure 7 (Fig. 7)). When looking at open-ended questions, students report a heterogenic feedback quality.

**Figure 8:**
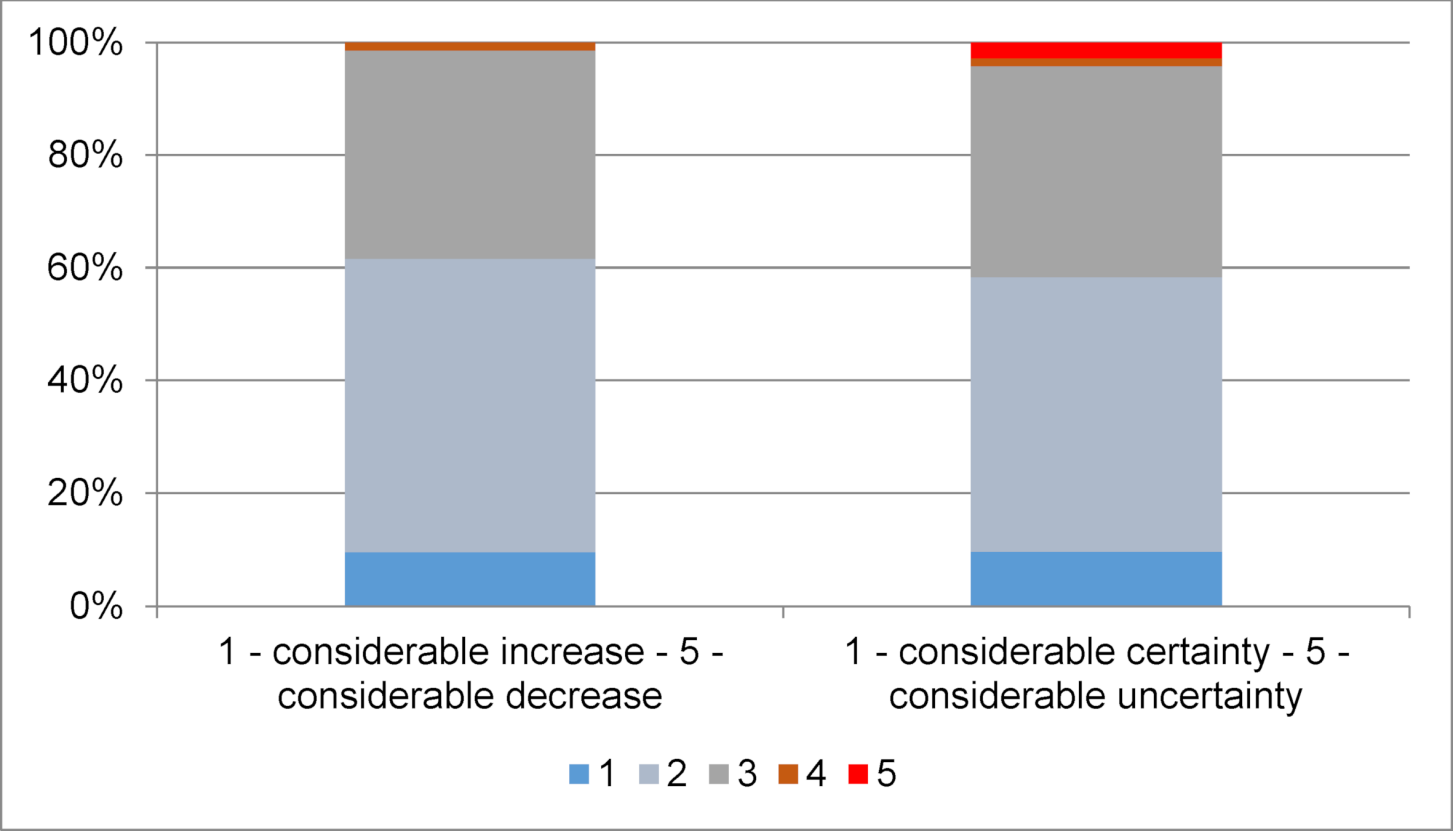
Competence in team communication after PBL (problem-based learning) course in emergency and acute care medicine with aspects of team communication, 4^th^ assessment point, cohort 1, n=72, mean age=24 years, 65% women, 35% men.

At T4, 60% of cohort 1 felt more confident in team communication, registering an improvement in personal team communication competence. About 10% report a strong improvement (see figure 8 (Fig. 8)). 

## 4. Discussion

After implementation of the Longitudinal Communication Curriculum, medical students at Leipzig University report an increase in communication skills after SP encounters. The combination of teaching communication and clinical examination skills in the Clinical Skills Course was judged as helpful; students feel that they benefit from the PBL courses on team communication. How self-rated communication skills develop over time will be investigated further on. These future results will be imminent for curricular adaptations.

Communication skills are assessed in years 3 and 4 in one OSCE station, respectively. It remains to be discussed if communication skills should be assessed in one way or another at each OSCE station in future, as is already the case in Basel [13].

Due to the COVID-19 pandemic, part IV of the Longitudinal Communication Curriculum was not conducted as originally intended. A screencast was produced; the planned PBL case, as well as SP encounters were cancelled. The first cohort could not participate in the complete Longitudinal Communication Curriculum. 

So far, SP encounters are mandatory in the 2^nd^ study year. Another compulsory SP encounter could help to train and assess students’ communication skills over time.

Implementation of feedback during OSCE was accompanied with concerns from some of the faculty members. On the other hand, student surveys show high satisfaction rates for receiving feedback during OSCEs. At the same time, there are student concerns regarding heterogenic feedback quality. Improving this will be a goal for future years.

Units of the Longitudinal Communication Curriculum were integrated into existing clinical courses of the medical curriculum. Adaptation of existing structures is a major challenge for communication skills integration so far.

We focused on the implementation of basic communication skills as well as team communication skills. To better acknowledge the role of "team manager" (CanMEDS, [9]) in the future, inter- and intraprofessional scenarios should be added. First experiences with an interprofessional elective course for students of the 9^th^ semester and midwifery students seem promising. At the same time, such projects are associated with high personnel costs and are difficult to integrate into the existing medical curriculum.

## 5. Conclusion

Like other faculties [13], [11], [14], [15], longitudinal basic communication skills are taught and assessed at the Medical Faculty of Leipzig University.

Quality assurance for SP, peer-student tutors and clinical colleagues need to be planned for ensuring a sustainable curriculum. We expect to implement the 4^th^ part of the curriculum next year.

For content development, an additional obligatory SP encounter as well as interprofessional teaching units shall be planned.

The curriculum is to be continued in the final year of medical education during hospital clerkships. So far, SP encounters for final-year medical students in their surgery rotation have been implemented. It would be desirable to teach communication skills to all students in their last year of study as well. 

## Profiles

**Name of the location:** Leipzig University, Medical Faculty 

**Subject/professional group: **Human medicine

**Number of learners per year or semester: **300-320 per year (every cohort)

**Has a longitudinal communication curriculum been implemented?** Yes

**In which semesters are communication and social skills taught?** 3., 4., 5., 7., 10.

**What teaching formats are used? **Lectures, courses, SP-conversations, tasks on video casts

**In which semesters are communication and social skills tested?**

- 5^th^ semester (1 OSCE station, communication skills integrated with clinical practical skills, relevant for passing exam, formative feedback for students in examination)
- 7^th^ semester (1 OSCE station, team communication skills integrated with clinical practical skills and formative feedback for students in examination)

**Which examination formats are used? **OSCE

**Who is responsible for the development and implementation?**

- working group longitudinal communication curriculum
- 1 academic worker for simulated patient programme and implementation of curriculum in cooperation with the professions involved in curriculum

## Current professional roles of the authors

- Anja Zimmermann, Dr. rer.medic.: psychologist and playwright. Since 2017 she is coordinator of the Longitudinal Communication Curriculum and SP-programme. She is responsible for role development, SP-training, qualification assurance, development and implementation of curriculum.
- Christoph Baerwald, Prof. Dr. med.: internist and lead in the section Rheumatology/Gerontology of the Medical Department III – Endocrinology, Nephrology, Rheumatology of University of Leipzig Medical Centre. He guides Clinical Skills Course for 5^th^ semester and is course director for Problem-Based-Learning in 10^th^ semester. He is contact person for the Longitudinal Communication Curriculum in 5^th^ and 10^th^ semester.
- Michael Fuchs, Prof. Dr. med.: otorhinolaryngologist and specialist for phoniatrics and paediatric audiologie. He is director of the Division of Phoniatrics and Audiology and of the Cochlea Implant Centre of University of Leipzig Medical Centre. He is responsible for coordination of teaching for otolaryngology and course director of Problem-Based-Learning in 10^th^ semester. He is contact person for the Longitudinal Communication Curriculum in 10^th^ semester.
- Heide Götze, PD Dr. rer. med.: psychologist and teaching coordinator for the Division of Medical Psychology and Medical Sociology. She is in the course of graduating from her studies in Heidelberg. For the Longitudinal Communication Curriculum she is responsible for communication courses in 3^th^ and 4^th^ semester.
- Christian Girbardt, Dr. med.: ophthalmologist and teaching coordinator for the Department of Ophthalmology. Concerning the Longitudinal Communication Curriculum he is responsible for courses in ophthalmology in the Clinical Skills Course that take place with SP encounters.
- Gunther Hempel, Dr. med., MME: anesthesiologist. He is consultant at the Interdisciplinary Operative Intensive Care Unit and coordinates teaching for Department of Anaesthesiology and Intensive Care. He graduated MME study in Heidelberg. For the Longitudinal Communication Curriculum, he is responsible for the PBL course in emergency care and intensive care medicine in the 7^th^ semester.
- Kai von Klitzing, Prof. Dr. med.: child psychiatrist. He is director of the Clinic for Psychiatry, Psychotherapy and Psychosomatics of Childhood and Adolescence at University Hospital Leipzig. He is also medical scientific director of the Department for Women and Child Medicine. He leads the Longitudinal Communication Curriculum task force and provides advice for all questions concerning curriculum.
- Daisy Rotzoll, PD Dr. med., MME (Bern): paediatrician and neonatologist. She is medical director of the Skills and Simulation Centre LernKlinik Leipzig. She leads the Longitudinal Communication Curriculum task force and provides advice for all questions concerning curriculum.

## Competing interests

The authors declare that they have no competing interests.
